# P-219. Zoster Infection Rivals HIV in Long-Term Neurologic and Cardiovascular Morbidity

**DOI:** 10.1093/ofid/ofaf695.441

**Published:** 2026-01-11

**Authors:** Ali Dehghani, George Yendewa

**Affiliations:** Department of Medicine, Case Western Reserve University School of Medicine, Cleveland, OH; Department of Medicine, Case Western Reserve University School of Medicine, Cleveland, OH

## Abstract

**Background:**

Despite antiretroviral therapy (ART), people with HIV (PLWH) remain at increased risk for dementia and cardiovascular disease, likely due to chronic inflammation. Herpes zoster (HZ) similarly contributes to neurovascular injury via viral neurotropism and vasculitis. Though both conditions share inflammatory sequelae, direct comparisons are lacking. We aimed to compare the time-to-event risks of dementia, major adverse cardiovascular events (MACE)—a composite of sudden cardiac death, stroke, myocardial infarction, and pulmonary embolism—and all-cause mortality between unvaccinated adults with HZ and ART-PLWH.

**Figure:**
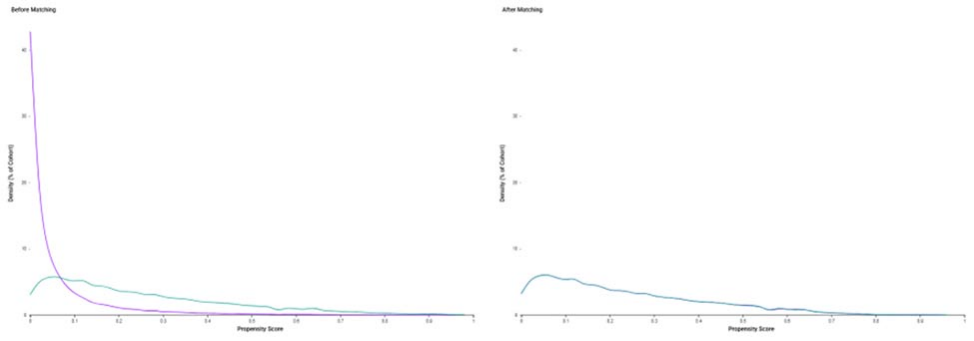
Propensity Score Density Function Before and After Matching

Distribution of propensity scores for Cohort 1 (purple); HZ and Cohort 2 (cyan); HIV, before (left) and after (right) 1:1 propensity score matching. Matching achieved excellent overlap and covariate balance across cohorts, indicating successful reduction of baseline confounding.

**Figure:**
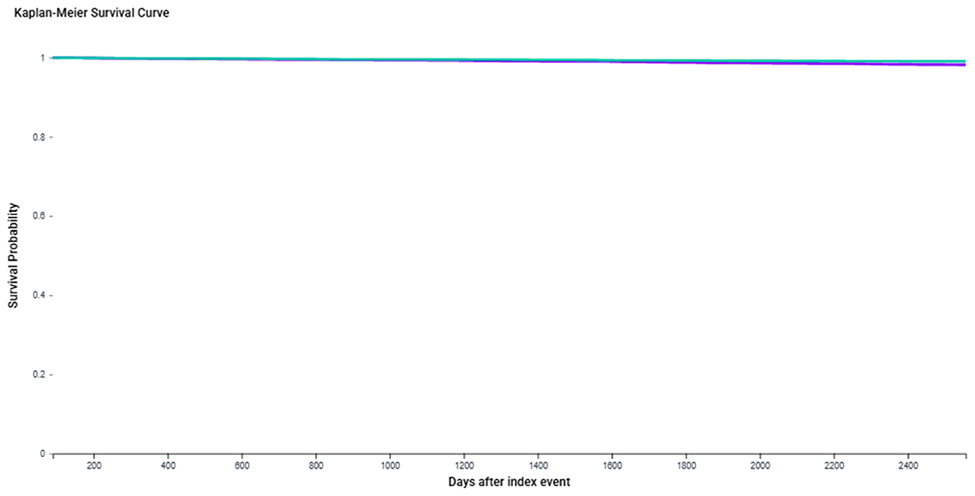
Kaplan-Meier Survival Curve for Dementia

Time-to-event analysis comparing the cumulative incidence of dementia between unvaccinated herpes zoster (HZ) patients (purple curve) and HIV-positive individuals on antiretroviral therapy (ART) (cyan curve) after 1:1 propensity score matching (n = 16,647 per group). Despite low absolute incidence, HZ was associated with a significantly higher hazard of dementia (HR = 1.78, 95% CI: 1.40–2.26, p = 0.0114). Survival curves begin to diverge within the first few years of follow-up, highlighting the potential long-term neuroinflammatory effects of HZ.

**Methods:**

We conducted a retrospective cohort study using the TriNetX platform, comparing unvaccinated adults with HZ to PLWH on ART, excluding individuals with prior HZ, HZ vaccination, hepatitis B or C, MACE, or dementia. Both cohorts (n=16,647 each) were 1:1 propensity-matched on demographics, psychiatric diagnoses, cardiometabolic comorbidities, and preventive care exposures (e.g., vaccines, statins, antihypertensives). All PLWH were on ART at index. Primary outcomes included dementia, MACE, and all-cause mortality. Secondary outcomes included psychiatric morbidity (anxiety, depression and schizophrenia), and Parkinsonism. Hazard ratios (HRs) and Kaplan-Meier analyses were used to assess outcome differences, with p < 0.05 considered statistically significant.

**Figure:**
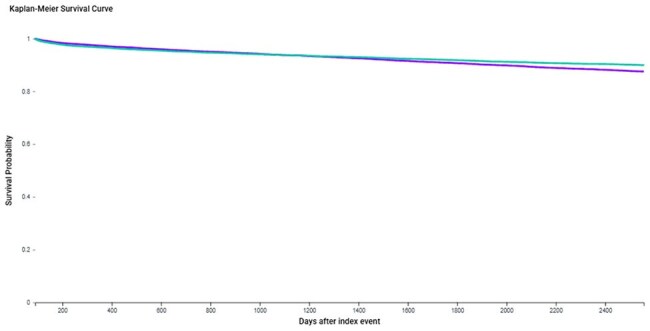
Kaplan-Meier Survival Curve for Major Adverse Cardiovascular Events (MACE)

Time-to-event analysis comparing the cumulative incidence of MACE in unvaccinated herpes zoster (HZ) patients versus HIV-positive individuals on antiretroviral therapy (ART), following 1:1 propensity score matching (n=16,647 per group). Median follow-up was 5.3 years (HZ) and 4.8 years (HIV). HZ was associated with a significantly higher hazard of MACE (HR 1.15, 95% CI 1.06–1.24, p = 0.0004) with divergence in survival probabilities over time.

**Figure:**
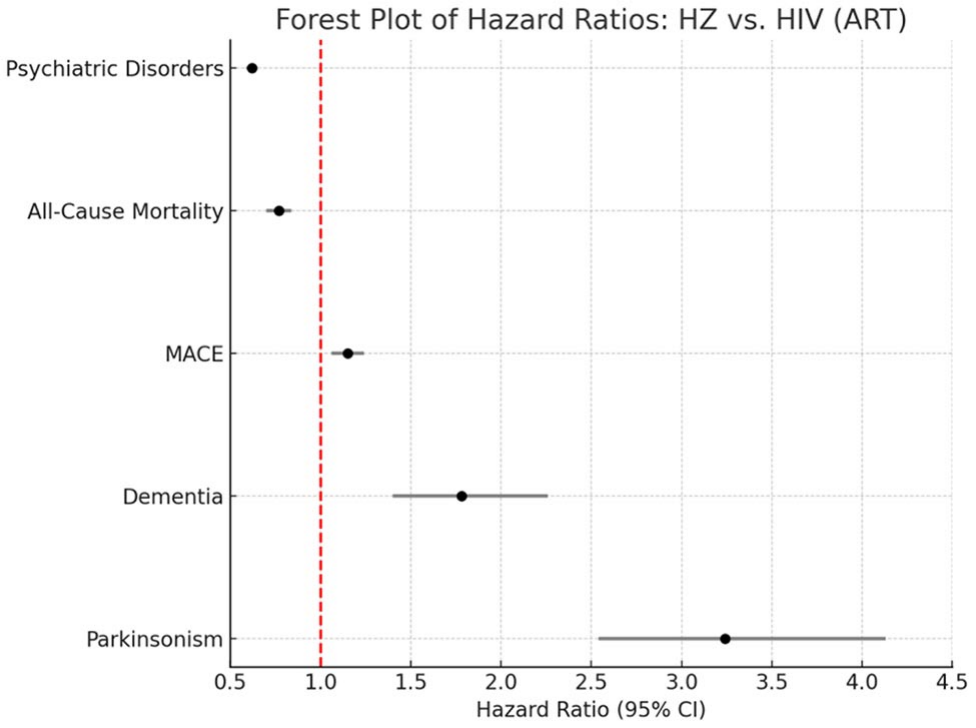
Forest Plot of Hazard Ratios for Clinical Outcomes Comparing Unvaccinated Herpes Zoster Patients to HIV-Positive Adults on ART

Forest plot displaying hazard ratios (HRs) with 95% confidence intervals (CIs) for primary and secondary outcomes among propensity-matched cohorts (n=16,647 each). Herpes zoster (HZ) infection was associated with significantly increased hazards of dementia (HR 1.78, 95% CI 1.40–2.26), major adverse cardiovascular events (MACE; HR 1.15, 95% CI 1.06–1.24) and Parkinsonism (HR 3.24, 95% CI 2.54–4.13), relative to HIV. In contrast, all-cause mortality (HR 0.77, 95% CI 0.70–0.84 and psychiatric disorders were more common in the HIV group (HR 0.62, 95% CI 0.59–0.65); p < 0.05.

**Results:**

After 1:1 matching (n=16,647 per group), mean age was 53 years, 67% were male, 38% were white and were followed from 90 days to 7 years post-index. The median follow-up was 5.3 years (HZ) and 4.8 years (HIV). HZ infection compared to HIV was associated with significantly higher hazards of dementia (HR 1.78, 95% CI 1.40–2.26), MACE (HR 1.15, 95% CI 1.06–1.24), and Parkinsonism (HR 3.24, 95% CI 2.54–4.13), compared to HIV; p < 0.05. In contrast, all-cause mortality (HR 0.77, 95% CI 0.70–0.84 and psychiatric disorders were more common in the HIV group (HR 0.62, 95% CI 0.59–0.65); p < 0.05.

**Conclusion:**

HZ infection was associated with greater long-term neurovascular morbidity than HIV, underscoring the importance of zoster prevention strategies. Expanded use of recombinant zoster vaccine may reduce neuroinflammatory complications in aging adults.

**Disclosures:**

All Authors: No reported disclosures

